# Tic disorders and allergic diseases: mechanistic links and the impact of allergy management – a narrative review

**DOI:** 10.3389/falgy.2026.1769483

**Published:** 2026-02-12

**Authors:** Lili Li, Wandong Hu, Ying Ren, Huan Zhang, Shushu Zhu, Tao Zhong, Hongwei Zhang

**Affiliations:** 1 Department of Neurology, Children’s Hospital Affiliated to Shandong University, Jinan, Shandong, China; 2 Department of Traditional Chinese Medicine, Children’s Hospital Affiliated to Shandong University, Jinan, Shandong, China

**Keywords:** allergic diseases, allergy management, neuroimmune crosstalk, tic disorders, Tourette syndrome

## Abstract

Tic disorders are childhood-onset neuropsychiatric conditions that frequently co-occur with allergic diseases, including asthma, allergic rhinitis, and atopic dermatitis. This narrative review maps the current clinical and mechanistic evidence linking allergic conditions to tic disorders and evaluates whether allergy focused interventions may modify tic severity. Across multiple epidemiological studies, allergic diseases are reported more often in children with tic disorders than in controls. However, direct clinical evidence supporting a canonical “central allergic response” within the brain (e.g., IgE-driven allergic effector mechanisms in the CNS) in primary tic disorders remains limited. The available literature instead more strongly supports indirect, peripheral, brain directed pathways. Peripheral inflammatory mediators may modulate the neurovascular unit and glial reactivity, thereby influencing cortico-striato-thalamo-cortical circuit excitability. Histamine, acting as both an immune mediator and a neuromodulator, may further intersect with dopaminergic signalling relevant to tic expression. In parallel, allergy related symptom burden, particularly sleep disruption and psychological stress, may contribute to tic exacerbation. Observational studies suggest that controlling allergic symptoms can be associated with reduced tic severity in some individuals, although certain anti allergic agents have been reported to coincide with tic worsening in selected cases. Overall, current findings support a model in which allergic conditions influence tic disorders primarily via immune signalling and symptom burden rather than through a direct central allergic mechanism. Allergy assessment and management may be considered in selected patients, but mechanistic studies and controlled trials are needed to clarify causality and guide evidence-based care.

## 1 Introduction

Tic disorders (TD) are a group of chronic neuropsychiatric conditions characterised by childhood onset of multiple, rapid, repetitive,apparently purposeless and involuntary motor and/or vocal tics. TD encompass provisional tic disorder, chronic motor or vocal tic disorder and Tourette syndrome (TS) (1, 2). The global prevalence of TD in children is estimated at approximately 0.3%–1.0%, with a pronounced male predominance (male-to-female ratio of approximately 4:1) (3). The prevalence of TS in children and adolescents is estimated to be close to 1% (4). Clinical manifestations range from simple tics, such as eye blinking, shoulder shrugging or throat clearing, to complex imitative behaviours, coprolalia and echolalia. TS also frequently co-occurs with psychiatric and behavioural disorders, including attention-deficit/hyperactivity disorder (ADHD), obsessive–compulsive disorder (OCD), self-injurious behaviour and anxiety (5, 6). These motor and behavioural symptoms contribute to social difficulties, learning impairments and bullying, imposing considerable psychological and economic burdens on affected families and reducing quality of life (7).

In parallel, allergic diseases such as asthma, allergic rhinitis and atopic dermatitis have become some of the most prevalent chronic illnesses in childhood worldwide (8–10). Current data indicate that approximately 10%–30% of individuals aged ≤18 years are affected by at least one allergic disease (11–13). Accumulating epidemiological evidence suggests a consistent association between TD and allergic diseases (14–16). In a case–control study of children and adolescents, 53.1% of patients with TD had at least one comorbid allergic disease compared with only 22.9% of controls, indicating a markedly higher comorbidity burden (17). An analysis of data from the US National Survey of Children's Health similarly showed that children with TD have significantly higher prevalences of asthma and allergic diseases, as well as a higher incidence of neuropsychiatric comorbidities, than children without TD (18). These findings suggest potentially shared or interacting pathophysiological pathways, such as immune dysregulation, neuroinflammation and genetic susceptibility, between TD and allergic diseases (19, 20). In addition, immune-mediated and autoimmune hypotheses, including infection-triggered symptom exacerbations in selected subgroups, have been increasingly discussed in TD, further motivating an integrated neuroimmune perspective (20). Notably, given the limited direct clinical evidence for a primary central allergic mechanism in TD/TS, this review focuses on peripheral immune signalling and symptom-burden pathways as plausible modulators of tic expression (19, 20). Consequently, clarifying whether and how allergy management influences TD outcomes has important clinical implications for refining comprehensive treatment strategies for TD.

## 2 Literature search strategy

This narrative review synthesizes evidence on the impact of allergy management on tic severity and prognosis in children and explores potential shared pathophysiological mechanisms linking allergies with tic symptoms. We searched PubMed, Embase, and Web of Science from database inception to 31 October 2023. Database-specific strategies combined controlled vocabulary (MeSH in PubMed; Emtree in Embase) with free-text keywords searched in titles/abstracts. The strategy comprised four concept blocks: (1) tic disorders/Tourette syndrome, (2) allergic diseases (allergy/allergic rhinitis/asthma/atopic dermatitis), (3) anti allergic medications/therapies (e.g., antihistamines, leukotriene receptor antagonists, corticosteroids, immunotherapy/anti-IgE), and (4) pediatric populations (child/adolescent/pediatric). Boolean operators (“AND”/“OR”) were used to combine terms, and equivalent syntaxes were adapted across databases. The complete, reproducible search strings for each database are provided in Supplementary Table S1.

Eligible publications comprised original research articles (randomized controlled trials, cohort studies, case–control studies), clinical trials, systematic reviews, meta-analyses and relevant narrative reviews. Studies were included if they addressed (i) associations between allergy treatment and tic severity, frequency or related comorbidities in individuals aged ≤18 years, or (ii) pathophysiological mechanisms underlying the comorbidity of tics and allergic diseases. Titles and abstracts were screened first, followed by full-text review to determine eligibility. Additional studies were identified through manual review of reference lists. Owing to heterogeneity in study design and outcome measures, a qualitative narrative synthesis was undertaken rather than a quantitative meta-analysis.

## 3 Potential pathophysiological mechanisms linking TS/TD to allergic diseases

Although TD/TS and allergic diseases exhibit markedly different clinical presentations, the substantial comorbidity reported in epidemiological studies suggests shared or interacting pathophysiological pathways. This section synthesises key clinical and mechanistic evidence linking allergic inflammation to tic disorders, with an emphasis on indirect peripheral to brain neuroimmune pathways that may modulate CSTC circuit excitability.

### 3.1 Immune–neuroinflammatory pathway

**Figure 1:**
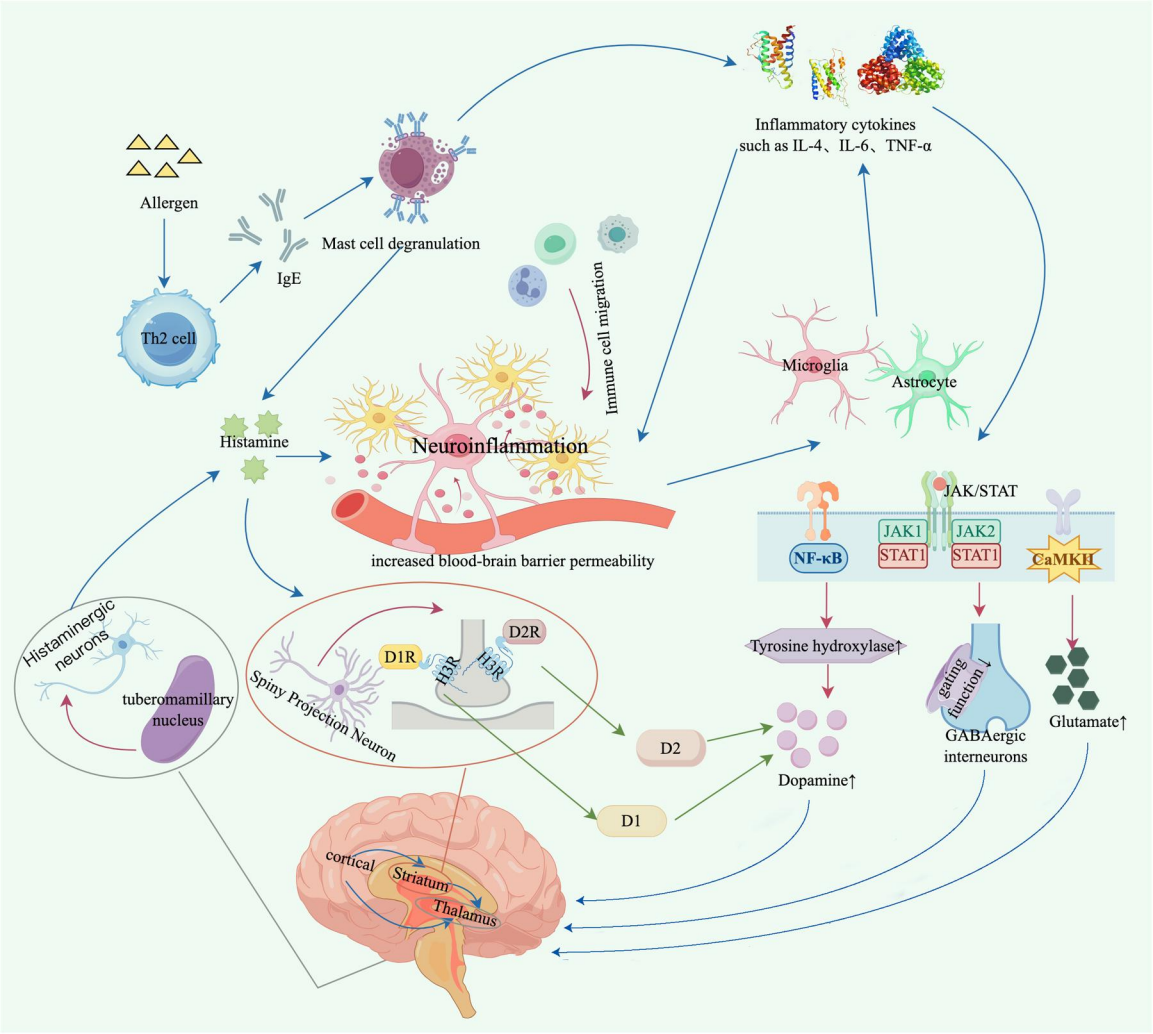
Schematic illustration of the hypothetical mechanisms linking allergic reactions and tic disorders. Allergen exposure activates Th2 cells and induces the production of allergen-specific IgE antibodies, which in turn can trigger mast-cell degranulation and the release of histamine and cytokines such as IL-4, IL-6, and TNF-α. These mediators may converge through two main pathways to increase the excitability of the CSTC circuit and thereby precipitate or aggravate tic symptoms. (1) Immune–neuroinflammatory pathway hypothesis: These cytokines can increase BBB permeability and activate the neurovascular unit, thereby facilitating immune to brain signalling signalling and promoting central inflammatory cascades under certain conditions. Within the brain, they activate microglia and astrocytes, driving further production of IL-4, IL-6, TNF-α, and other pro-inflammatory cytokines and amplifying chemokine signalling and endothelial activation. Together, these processes exacerbate BBB disruption and neuroinflammation through self-reinforcing neurovascular and glial inflammatory signalling. The resulting inflammatory milieu up-regulates tyrosine hydroxylase via NF-*κ*B, JAK/STAT, and CaMKII signaling pathways and enhances dopamine release, while impairing the gating function of GABAergic interneurons and interfering with glutamate clearance. This disturbance of the excitatory–inhibitory balance within the CSTC circuit facilitates the premature release or amplification of inappropriate motor programs—abnormal motor patterns that are not adequately suppressed—thereby inducing or worsening tic symptoms. (2) Histamine–dopamine axis hypothesis: Histamine generated during allergic reactions increases BBB permeability by disrupting tight junction proteins, thereby promoting neurovascular-unit activation and immune to brain signalling signalling. Together with histamine released from histaminergic neurons in the tuberomammillary nucleus and from meningeal and perivascular mast cells undergoing degranulation in neuroinflammatory contexts, this histaminergic input amplifies neuroinflammation through inflammatory cascades initiated by the immune–neuroinflammatory pathway, creating a permissive environment for dopaminergic imbalance. In parallel, histamine acts on H3R–D1R and H3R–D2R transmembrane complexes on striatal medium spiny projection neurons, shifting the balance between D1- and D2-mediated dopaminergic signaling. This shift lowers the inhibitory threshold of the CSTC circuit so that less inhibitory input is required to trigger neuronal activity, thereby increasing the likelihood of tic expression.

An increasing body of evidence indicates that inflammatory responses represent one of the key pathophysiological mechanisms underlying the development of TD/TS (21–23). Moreover, bidirectional interactions between the nervous and immune systems have been demonstrated (24), and may provide a conceptual basis for shared immuno-neuroinflammatory pathways between TD/TS and allergic diseases (Figure 1).

#### 3.1.1 Immune activation and inflammatory cytokine expression

Allergic diseases are primarily characterized by a T-helper-2 (Th2)-type immune response. Allergen-specific IgE antibodies can trigger mast-cell degranulation, leading to the release of histamine and multiple type 2 cytokines, including IL-4, IL-5 and IL-13; in chronic allergic inflammation and epithelial injury, mast cells and other innate immune cells may also contribute broader inflammatory mediators such as IL-6 and TNF-α (24, 25). Epithelial “alarmins” released during allergic inflammation, such as TSLP, IL-33 and IL-25, can amplify innate and myeloid activation, which may broaden the cytokine milieu beyond type 2 mediators and contribute to IL-1β, IL-6 or TNF-α signalling (24). Studies have reported significantly elevated peripheral concentrations of pro-inflammatory markers such as TNF-α, IL-6 and IL-4 in children with TD, suggesting that a low-grade inflammatory state may be associated with fluctuations in TD/TS severity (26, 27).

One proposed mechanism involves these cytokines increasing blood–brain barrier (BBB) permeability and activating microglia and astrocytes (28, 29). At the molecular level, cytokine signalling can impair BBB function through neurovascular-unit activation, including NF-κB, MAPK and JAK/STAT pathways, with downstream effects on tight-junction proteins including claudin-5, occludin and ZO-1, cytoskeletal organisation, and endothelial activation through adhesion-molecule upregulation and matrix remodelling, thereby increasing paracellular leakiness (30, 31). For example, IL-1β has been shown to disrupt BBB integrity by suppressing astrocyte-derived sonic hedgehog signalling, thereby weakening tight-junction support (30). Once activated, these cells release additional pro-inflammatory chemokines and amplify neurovascular inflammation and endothelial activation; in selected inflammatory contexts this may facilitate leukocyte adhesion or trafficking, although the extent of immune-cell infiltration into brain parenchyma in primary TD/TS remains uncertain (30, 32). Here, immune to brain signalling signalling refers to neurovascular-unit activation and glial priming; it does not imply established, robust leukocyte infiltration into brain parenchyma in primary TD/TS (31). Furthermore, inflammatory injury of the striatal microglia–dopaminergic-neuron crosstalk has been implicated in the development of TS (33).

#### 3.1.2 Effects of neuroinflammation on neural circuits

Current research suggests that TD/TS may result from inhibitory dysfunction within the cortico-striato-thalamo-cortical (CSTC) circuit. A core pathophysiological feature is relative hyperactivity of the striatal “direct pathway” (D1 receptor-mediated) together with relative insufficiency of the “indirect pathway” (D2 receptor-mediated). This imbalance leads to premature release of motor programs that would normally be suppressed, ultimately triggering tic symptoms (34, 35).

Peripheral pro-inflammatory factors such as IL-1β, IL-6 and TNF-α can increase BBB permeability, thereby enhancing neurovascular-unit signalling and facilitating immune to brain signalling communication that may promote central inflammatory cascades under certain conditions, with direct CNS entry being context-dependent rather than assumed (30, 31). Activated microglia and astrocytes then release inflammatory mediators that may upregulate tyrosine hydroxylase via signalling cascades including NF-κB, JAK/STAT and CaMKII, thereby promoting dopamine synthesis and release. In parallel, neuroinflammation may impair the gating function of γ-aminobutyric-acid (GABA)-ergic interneurons and disrupt glutamate clearance. Together, these processes disturb the excitatory–inhibitory balance of the CSTC circuit (33, 34, 36), facilitating the premature release or amplification of inappropriate motor programs and thereby inducing or exacerbating TD/TS symptoms.

### 3.2 Histamine–dopamine axis hypothesis

Histamine acts not only as a key mediator of allergic reactions but also as an important neurotransmitter in the brain (37, 38). In this hypothesis, histamine is discussed as a convergence node linking allergy-associated peripheral mediator release and neurovascular-unit activation with central histaminergic modulation of striatal dopamine signalling relevant to tic pathophysiology (37–39). During allergic reactions, IgE-mediated degranulation of mast cells leads to rapid release of histamine and other mediators, including cytokines such as IL-1, IL-6 and TNF-α. This cascade increases BBB permeability by disrupting tight-junction proteins and upregulates adhesion-molecule expression, thereby promoting neurovascular-unit activation and immune to brain signalling signalling; the extent to which this results in leukocyte trafficking is context-dependent (31, 40).

In addition to histamine released by histaminergic neurons in regions such as the tuberomammillary nucleus of the hypothalamus, mast cells in the meninges and perivascular spaces can also be activated in neuroinflammatory contexts, leading to further local histamine release. This, in turn, may promote pro-inflammatory polarization of microglia and astrocytes and may affect synaptic homeostasis via pathways such as JAK/STAT, NF-κB and CaMKII, including modulation of glutamate clearance, inhibitory gating and tyrosine hydroxylase expression (31, 40, 41). These changes create a neuroinflammatory environment that may favour increased striatal dopamine activity (41).

Histamine H3 receptors (H3R) are highly enriched in striatal spiny projection neurons and can assemble into heteromeric complexes with D1 and D2 dopamine receptors, enabling receptor–receptor allostery within the same membrane microdomain (42–44). In D1R–H3R heteromers, heteromerization can switch canonical D1R coupling from Gαs to Gαi, blunting cAMP signaling and permitting robust MAPK engagement; importantly, antagonism of either protomer can “cross-block” heteromer-driven signaling, consistent with conformational coupling across the complex (43, 44). In D2R–H3R heteromers, H3R activation can allosterically reduce D2R agonist affinity and re-tune D2R-dependent intracellular cascades, including a β-arrestin2–Akt–GSK3β signaling axis in D2R-SPNs (42). This heteromer-specific cross-talk provides a mechanistic basis by which H3R can gate D1/D2 signaling integration and dopamine-related outputs within CSTC circuitry; when dysregulated, it may weaken inhibitory gating and facilitate tic expression or exacerbation (45).

### 3.3 Genetic susceptibility

Both TD/TS and allergic diseases have distinct yet partially overlapping genetic backgrounds. Several population-wide genome-wide association studies (GWAS) have demonstrated positive genetic correlations between TD/TS and allergic diseases, suggesting partial overlap of risk loci and shared genetic backgrounds at the population level (14, 46, 47). GWAS and gene-set enrichment analyses of TS have revealed a dual genetic basis: on the one hand, associations with synaptic plasticity and synaptosomal function; and on the other, close links to immune, inflammatory and cell-adhesion-molecule pathways (14, 48, 49).

The *CDH26* gene is of particular interest, as it lies within a TS linkage region and shows differential methylation in epigenome-wide association studies (EWAS) of nasal epithelial cells from individuals with allergic disease. Functional studies further indicate that CDH26 exerts a dual mechanism of action at the mucosal interface: as an epithelial cadherin, it mediates Ca^2+^-dependent homotypic adhesion and associates with catenin-family partners to support epithelial cell–cell contact and barrier architecture; in airway epithelium, CDH26 is further implicated in maintaining cortical actin organization and apicobasal polarity, consistent with a role in preserving mucosal-barrier integrity (49, 50). In parallel, CDH26 functions as an *α*4/αE integrin-binding epithelial ligand, and recombinant CDH26-Fc can suppress human CD4^+^ T-cell activation and reduce IL-2 secretion, supporting a direct immunoregulatory role that complements its barrier-maintenance function (49). In addition, rare familial studies have shown that loss-of-function mutations in the *HDC* gene, which encodes L-histidine decarboxylase (the rate-limiting enzyme in histamine biosynthesis), can cause TS, highlighting the critical role of histaminergic neurotransmission in the pathogenesis and modulation of TS and tic symptoms (51, 52).

Moreover, studies of both TD/TS and allergic diseases have pointed to genetic abnormalities in inflammatory-factor axes (e.g., TNF-α, IL1RN) (14), suggesting shared inflammatory-factor genetic mechanisms. Nevertheless, most robust evidence currently derives from genetic correlations, functional overlap in specific pathways or gene sets (such as GABAergic signalling and immune-related cell-adhesion molecules) and rare high-impact variants (e.g., *HDC*) (14, 46, 48, 51–53). Establishing stable causal associations between individual common single-nucleotide polymorphisms and disease phenotypes will require further validation using approaches such as polygenic-risk scores, cross-phenotype colocalisation analyses and systematic functional experiments in larger samples.

### 3.4 Epigenetic regulation

Patients with allergic diseases exhibit specific DNA-methylation patterns in nasal and airway epithelia that can distinguish distinct allergic phenotypes. Differentially methylated genes are primarily enriched in epithelial-barrier and immune-regulation pathways (e.g., *CDH26* and *CDHR3*), confirming significant epigenetic reprogramming at the peripheral immune–epithelial barrier interface (53). Although TD/TS studies to date have been limited by small sample sizes, EWAS consistently reveal disease associated methylation abnormalities, further supporting an epigenetic contribution to immune–neurodevelopmental pathways (54, 55).

Group A β-haemolytic streptococcal infection is thought to trigger autoimmune responses via molecular mimicry and to mediate dysregulation of immune–neuro logical pathways through epigenetic reprogramming, illustrating how epigenetic mechanisms may operate in TD/TS (56, 57). On this basis, it can be hypothesised that allergy-induced immune activation and environmental exposure, including ambient air pollution (e.g., PM_2.5_ or PM_10_, NO_2_ and O_3_), influence gene expression via shared epigenetic mechanisms, such as DNA methylation, histone modifications and non-coding RNA regulation (58–60). Supporting this, a large multi-tissue epigenomic analysis in children (placenta, cord blood and nasal or airway-related specimens) identified pollutant- and window-specific DNA methylation differences at numerous asthma-or allergy-relevant genes in relation to prenatal PM_2.5_, NO_2_, O_3_ exposure (58), and independent birth-cohort data link prenatal particulate exposure to placental methylation signatures (including Notch and neurodevelopment-related pathways) associated with childhood asthma susceptibility (59). Importantly, air pollutants can also reach the brain and have been increasingly connected to neurodevelopmental and neurodegenerative outcomes, with epigenetic alterations proposed as mediators linking inhalational exposure to neuroinflammation and synaptic pathways (61, 62). However, these observational data cannot establish causality and may be influenced by confounding and comorbidities. These mechanisms may not only reshape peripheral immune states but also modulate central nervous system inflammatory thresholds via cell-adhesion-molecule-related pathways. In genetically susceptible individuals, such changes may further amplify CSTC-circuit dysfunction by interacting with GABAergic inhibitory imbalance (14, 48, 49, 53–55, 63).

## 4 Allergy management strategies and their impact on TD outcomes

Standard care for TD/TS typically begins with psychoeducation and evidence-based behavioural interventions as first-line approaches; pharmacological therapy is considered when tics are moderate to severe and/or associated with clinically meaningful functional impairment (34). Given the frequent co-occurrence of allergic diseases in TD/TS and accumulating evidence for interacting neuroimmune mechanisms, assessment and management of comorbid allergic disease may represent an adjunctive component of comprehensive care for selected patients. This section summarises current allergy management strategies and critically appraises the available evidence regarding their associations with tic-related outcomes.

### 4.1 Effects of environmental control and allergen avoidance on TD outcomes

Environmental factors such as house dust mites, pollen and pet dander are major allergens that trigger allergic reactions. Altered IgE profiles and allergen sensitisation have been reported in children with tic disorders, raising the possibility that allergic inflammation may contribute to tic fluctuation in susceptible individuals (15). Cross-sectional case–control studies have reported associations between allergic comorbidity and greater clinical symptoms and difficulties in emotional and social communication in patients with TD (64, 65). Measures such as improving living environments, using hypoallergenic bedding, regularly cleaning air-filtration systems and avoiding pet exposure can reduce allergen burden and allergic reactions (66). From a mechanistic perspective, lowering allergen exposure may attenuate allergic inflammation and downstream immune signalling that could interact with tic-relevant neural circuits.

However, high-quality case–control studies or randomized clinical trials specifically testing whether environmental control and allergen avoidance can improve tic outcomes are lacking. Only a few small case series have suggested that systematic anti allergic management, combining pharmacologic and environmental interventions, may be associated with improvement in tic symptoms, but these studies are limited by small sample sizes, confounding interventions and low evidence quality (67). Notably, the contribution of environmental control alone cannot be disentangled in such combined-management designs.

Allergic rhinitis is commonly associated with poor sleep quality, and the prevalence of sleep disorders is significantly increased in patients with TD and TS (68). Therefore, alleviating allergic rhinitis may indirectly contribute to improved tic control by improving sleep disturbances caused by allergic symptoms. Moreover, tic severity in TS has been reported to be higher in spring and summer compared with autumn and winter, suggesting that seasonal environmental factors such as allergens and temperature changes may influence disease activity through specific physiological pathways (69). These observations are compatible with an environmental contribution to symptom variability, although the precise biological pathways remain to be elucidated.

In summary, environmental control and allergen-avoidance strategies may hold promise for improving TD symptoms, but the current level of evidence and certainty remains insufficient. Well-designed prospective studies, ideally with stratification by allergic phenotype and standardised tic outcome measures, are needed to validate these observations.

### 4.2 Impact of antihistamine and other anti allergic therapies on TD outcomes

Antihistamines and other anti allergic drugs are the mainstay treatments for allergic diseases and allergic inflammation. Most of these agents act by preventing the release of inflammatory mediators or by antagonising their effects on target cells (70). Based on their mechanisms of action, anti allergic medications can be broadly categorised into five classes: histamine H1-receptor antagonists, leukotriene antagonists, thromboxane A2 inhibitors, T-helper-cytokine inhibitors and mediator-release inhibitors. Corticosteroids exert their anti allergic effects primarily by suppressing T-helper cytokine production and inflammatory mediators (70, 71).

Despite their widespread use, studies directly examining the impact of anti allergic drugs on tic outcomes remain limited. Case reports have suggested that intranasal corticosteroids such as budesonide and fluticasone propionate may induce or exacerbate tic symptoms, with rapid resolution after discontinuation (72, 73). Given the nature of case reports, these observations support a temporal association but do not establish causality. In a nationwide population-based study, prescriptions of leukotriene receptor antagonists (LTRAs), primarily montelukast, were associated with a higher incidence of TS in children with asthma, allergic rhinitis or atopic dermatitis (74). Another population-based study found significantly higher rates of neurobehavioural disorders, including tic disorders, anxiety disorders and conduct disorders, among 5- to 10-year-old children exposed to the first-generation antihistamine hydroxyzine (75). As is common in observational pharmacoepidemiologic research, potential residual confounding, indication bias, and diagnostic misclassification may partly explain these observed associations. A summary of human clinical studies on the effects of anti allergic medications or therapies on TD or TS is provided in Supplementary Table S2.

Overall, evidence regarding the effects of anti allergic medications on TD/TS outcomes remains scarce and heterogeneous. Current data primarily suggest that certain agents have been reported to coincide with tic and other neuropsychiatric symptoms in susceptible children. Therefore, clinicians should exercise caution when prescribing anti allergic medications to paediatric patients with comorbid allergies and tic disorders, carefully weighing potential risks and benefits and monitoring neurobehavioural symptoms over time.

### 4.3 Impact of immunotherapy on allergic diseases with concurrent TD

In recent years, immunomodulatory therapies have led to major advances in the treatment of allergic diseases, particularly through the use of allergen immunotherapy and targeted biologics directed at specific immune pathways. Examples include the anti-IgE antibody omalizumab; anti-IL-5 antibodies mepolizumab and reslizumab; the IL-5-receptor-alpha antagonist benralizumab; the IL-4-receptor-alpha antagonist dupilumab; and anti-thymic-stromal-lymphopoietin antibodies, all of which have demonstrated efficacy with acceptable safety profiles in selected populations (76, 77).

Retrospective studies suggest that systemic anti allergic treatment, including immunotherapeutic approaches such as sublingual and subcutaneous immunotherapy and omalizumab, may improve clinical outcomes in children with refractory TD and ADHD (67). Nevertheless, it remains difficult to disentangle the direct vs. indirect effects of immunomodulation on tic outcomes, given the complex interplay between immune status, allergic symptom control and neuropsychiatric comorbidities. In a randomized controlled trial of paediatric asthma, one case of drug-related moderate tic disorder was reported as a serious adverse event in the omalizumab treatment group, suggesting that caution is warranted when using omalizumab in patients with both allergic conditions and TD (78). Notably, this was a single event in an asthma trial, and causality cannot be inferred from this observation alone. In addition, a small double-blind, placebo-controlled study found that intravenous immunoglobulin was ineffective for TD treatment, although allergic factors were not explicitly addressed (79).

Overall, clinical trials of immunomodulatory therapies specifically targeting TD are limited, and robust large-scale, multicentre randomized controlled studies are lacking. Furthermore, immunomodulatory therapies are costly and may carry infection-related risks in some settings and require careful patient selection and monitoring. Although these treatments show substantial potential for managing allergic diseases complicated by TD, further research is required to clarify their efficacy, safety and optimal indications in this population.

## 5 Limitations of the current evidence and future directions

This review summarises potential mechanisms underlying the comorbidity of allergic diseases and TD/TS, as well as the impact of allergy management on tic-related outcomes. Several important limitations of the existing literature should be acknowledged.

First, with regard to mechanistic research, current studies suggest that immune-system dysregulation, neuroinflammation, histaminergic–dopaminergic circuits, genetic susceptibility and epigenetic mechanisms may underlie the co-occurrence of allergic diseases and TD/TS. However, most of these mechanisms remain at the hypothesis-generating stage and lack robust molecular-biological and neuroimmunological validation.

Second, in terms of the clinical impact of allergy management on tic-related outcomes, most available studies are small observational studies characterised by high heterogeneity in design, exposure definition and outcome measures, as well as potential publication bias. Consequently, key questions in this field remain largely unexplored. Additional challenges include confounding by indication, concurrent psychotropic and anti allergic medication use, comorbid neuropsychiatric conditions, and the natural waxing and waning course of tics, all of which complicate causal inference.

Future research should prioritise elucidation of the interactive mechanisms between allergy-related immune inflammation and neural circuits, integrating the combined effects of genetic and environmental factors to clarify the mechanisms of comorbidity between allergic diseases and TD/TS. Such work will provide a foundation for developing personalised, mechanism-based treatment strategies. Methodologically, well-powered longitudinal cohorts with harmonised phenotyping, standardised tic severity measures, and careful documentation of allergic status and medication exposure are needed. Multimodal approaches integrating peripheral biomarkers, neuroimaging, and genetic or epigenetic profiling may help identify biologically meaningful subgroups and testable pathways. In addition, the impact of allergy management on tic-related outcomes requires further investigation through large-scale, multicentre randomized controlled trials and prospective cohort studies in order to generate high-quality evidence. Where feasible, trials should consider stratification by allergic phenotype and prespecified outcomes that distinguish tic severity from broader neurobehavioural symptoms.

## 6 Conclusion

Existing epidemiological and clinical studies indicate a clear consistent association between allergic diseases and TD/TS. Immune-inflammatory responses, neuroimmune interactions and genetic as well as environmental factors may collectively contribute to the pathogenesis of both allergic diseases and TD/TS, and they offer novel targets for clinical intervention. Current evidence suggests that effective, comprehensive allergy management may alleviate tics alongside allergic symptoms in some children; however, some antiallergic medications have the potential to induce or exacerbate tic symptoms and should be used cautiously in patients with comorbid TD/TS and allergies.

In routine practice, pediatricians should integrate allergy-oriented evaluation into TD care by systematically screening for common allergic comorbidities (especially allergic rhinitis/conjunctivitis, asthma, and atopic dermatitis), identifying symptom triggers and recent medication exposures, and explicitly assessing sleep disturbance, fatigue, and stress that can amplify tic severity. Management should prioritize guideline-based control of allergic inflammation (foundational non-pharmacologic measures and first-line anti-inflammatory therapies as appropriate), while minimizing polypharmacy and monitoring tic changes after treatment initiation or dose adjustments to distinguish medication-related effects from the natural waxing and waning course of TD. Overall, the potential benefit of allergy management for tic improvement is likely indirect through relief of allergic symptoms and related sleep/stress triggers, rather than direct modification of core TD circuitry. Further well-designed studies are needed to validate mechanisms and clinical effects; meanwhile, incorporating structured allergy assessment, prudent medication selection, and timely referral for uncontrolled allergic disease may help deliver more personalized, comprehensive care for affected children and their families.
