# Supplementary material for: Tic disorders and allergic diseases: mechanistic links and the impact of allergy management – a narrative review
Source: Front Allergy. 2026 Feb 12;7:1769483. doi: 10.3389/falgy.2026.1769483 (PMC12935891; doi:10.3389/falgy.2026.1769483)
Supplement: Supplementary file 1 [file Table1.docx]

Supplementary Table 1. Full search strategies

| **Database** | **Search strategy (verbatim)** |
| --- | --- |
| PubMed | (("Tic Disorders"[Mesh] OR "Tourette Syndrome"[Mesh] OR tic*[tiab] OR tourette*[tiab] OR "tic disorder*"[tiab] OR "Tourette syndrome"[tiab]) AND ("Allergy and Immunology"[Mesh] OR "Allergic Rhinitis"[Mesh] OR "Asthma"[Mesh] OR "Dermatitis, Atopic"[Mesh]  OR allerg*[tiab] OR "allergic rhinitis"[tiab] OR asthma*[tiab] OR "atopic dermatitis"[tiab] OR eczema*[tiab]) AND ("Histamine H1 Antagonists"[Mesh] OR antihistamin*[tiab] OR "histamine antagonist*"[tiab]  OR "Leukotriene Antagonists"[Mesh] OR montelukast[tiab] OR "leukotriene receptor antagonist*"[tiab]  OR corticosteroid*[tiab] OR "intranasal corticosteroid*"[tiab] OR budesonide[tiab] OR fluticasone[tiab]  OR immunotherap*[tiab] OR "allergen immunotherapy"[tiab] OR omalizumab[tiab] OR "anti-IgE"[tiab]) AND ("Child"[Mesh] OR "Adolescent"[Mesh] OR child*[tiab] OR pediatric*[tiab] OR paediatric*[tiab] OR adolescent*[tiab])) |
| Embase | ('tic disorder'/exp OR 'tourette syndrome'/exp OR tic*:ti,ab,kw OR tourette*:ti,ab,kw OR 'tic disorder*':ti,ab,kw) AND ('allergy'/exp OR 'allergic rhinitis'/exp OR 'asthma'/exp OR 'atopic dermatitis'/exp OR allerg*:ti,ab,kw OR 'allergic rhinitis':ti,ab,kw OR asthma*:ti,ab,kw OR 'atopic dermatitis':ti,ab,kw OR eczema*:ti,ab,kw) AND ('antihistaminic agent'/exp OR antihistamin*:ti,ab,kw OR 'leukotriene receptor antagonist'/exp OR montelukast:ti,ab,kw OR 'leukotriene receptor antagonist*':ti,ab,kw OR 'corticosteroid'/exp OR budesonide:ti,ab,kw OR fluticasone:ti,ab,kw OR 'intranasal corticosteroid*':ti,ab,kw OR 'allergen immunotherapy'/exp OR immunotherap*:ti,ab,kw OR omalizumab:ti,ab,kw OR 'anti-IgE':ti,ab,kw) AND ('child'/exp OR 'adolescent'/exp OR child*:ti,ab,kw OR pediatric*:ti,ab,kw OR paediatric*:ti,ab,kw OR adolescent*:ti,ab,kw) |
| Web of Science | TS=((tic* OR tourette* OR "tic disorder*" OR "Tourette syndrome") AND (allerg* OR "allergic rhinitis" OR asthma* OR "atopic dermatitis" OR eczema*) AND (antihistamin* OR "histamine antagonist*" OR montelukast OR "leukotriene receptor antagonist*" OR corticosteroid* OR "intranasal corticosteroid*" OR budesonide OR fluticasone OR immunotherap* OR "allergen immunotherapy" OR omalizumab OR "anti-IgE") AND (child* OR pediatric* OR paediatric* OR adolescent*))  Timespan: inception-31 October 2025; Indexes: SCI-EXPANDED, SSCI (modify as applicable) |
