# Supplementary material for: Tic disorders and allergic diseases: mechanistic links and the impact of allergy management – a narrative review
Source: Front Allergy. 2026 Feb 12;7:1769483. doi: 10.3389/falgy.2026.1769483 (PMC12935891; doi:10.3389/falgy.2026.1769483)
Supplement: Supplementary file 2 [file Table2.docx]

# Supplementary Table 2:Summary of human clinical studies on the effects of anti-allergic medications or therapies on TD/TS

| **Drug class** | **Drug** | **Study design** | **Effect on tics** | **Key findings** | **Reference** |
| --- | --- | --- | --- | --- | --- |
| **First-generation H1 antihistamines** | Hydroxyzine | Retrospective longitudinal cohort study | Negative (increased risk) | Early/repeated hydroxyzine prescriptions were associated with a higher risk of receiving a tic disorder diagnosis by age 10: repeated prescriptions vs single prescription, OR ≈ 1.55 (95% CI 1.23–1.96). | Gober HJ et al.(1) |
|  | Promethazine | Case report | Negative (induced/exacerbated) | An adolescent developed tics/movement-disorder manifestations after promethazine exposure, highlighting the potential CNS adverse-effect profile of first-generation antihistamines. | Akinsiku F et al.(2) |
| **Second-generation H1 antihistamines** | Cetirizine | Clinical trial (ETAC: RCT; safety-focused) | Neutral/insufficient (not tic-outcome trial) | In a long-term safety trial in infants/young children, overall safety was acceptable; however, TD/TS was not a primary endpoint, so tic-related conclusions are limited to indirect “no clear signal” evidence. | Simons FE（ETAC）(3) |
|  |  | Case series/case descriptions (refractory TD/TS comorbid with allergic rhinitis) | Positive (improved; confounded by combination therapy) | Within a standardized allergic rhinitis management package (including cetirizine), follow-up suggested improvement/resolution of tics; however, concomitant intranasal steroids, LTRAs, immunotherapy, and adjustments of anti-tic medications preclude attribution to cetirizine monotherapy. | Yu RL et al.(4) |
| **Leukotriene receptor antagonists (LTRAs)** | Montelukast | Nationwide population-based cohort study | Negative (risk ↑) | Taiwan NHIRD cohort: LTRA users (predominantly montelukast) had a higher TS risk than non-users; aHR ≈ 1.376 (95% CI 1.232–1.536). | Tsai ML et al.(5) |
|  |  | Population-based cohort study | Negative (higher risk with longer exposure) | In a pediatric asthma cohort, overall neuropsychiatric outcomes were not significantly increased; however, among children aged 6–15 years, use >63 days was associated with a higher risk of “tics/TS” (≈2.6-fold in girls; ≈1.8-fold in boys). | Lei WT et al.(6) |
|  |  | Case series/case descriptions (refractory TS comorbid with AR/asthma) | Positive (improved; confounded by combination therapy) | Montelukast was included in a multimodal AR/asthma management regimen; follow-up suggested improvement/resolution of tics, but concomitant intranasal steroids, antihistamines, (in some cases) anti-IgE therapy, and surgery/immunotherapy prevent isolation of a montelukast-specific effect. | Yu RL et al.(4) |
| **Intranasal/inhaled corticosteroids** | Fluticasone propionate | Case report | Negative (induced/exacerbated) | New-onset or worsening tics were reported in a child receiving fluticasone; symptoms resolved after discontinuation (signal for tic-related adverse effects). | Trau SP et al.(7) |
|  |  | Case report/letter (case signal) | Negative (induced/exacerbated) | Case signal suggesting tics emerged or worsened in pediatric asthma patients during fluticasone treatment. | Steele M, Rosner J et al.(8) |
|  |  | Meeting abstract/case report | Negative (induced/exacerbated) | A report described motor tics and mood changes following intranasal fluticasone (case-level evidence). | D’Silva N, Lindsay D et al.(9) |
|  | Mometasone furoate nasal spray | Case series/case descriptions (refractory TS comorbid with AR) | Positive (improved; confounded by combination therapy) | Mometasone was part of standardized AR management; follow-up suggested tic improvement/resolution, but concurrent cetirizine/montelukast/nasal irrigation/immunotherapy and anti-tic medication adjustments preclude attribution to mometasone alone. | Yu RL et al.(4) |
|  | Budesonide (with formoterol) | Case series/case descriptions (refractory TS comorbid with asthma + AR) | Positive (improved; confounded by combination therapy) | Budesonide/formoterol inhalation was included alongside standardized AR management and (in some cases) omalizumab; tics improved/resolved, but the independent effect of inhaled corticosteroids cannot be separated. | Yu RL et al.(4) |
| **Systemic corticosteroids**  **Systemic** | Prednisolone | Case series (2 cases) | Negative (exacerbated) | Two patients with “subclinical” tics experienced tic exacerbation during prednisolone treatment for other indications, suggesting systemic steroids may precipitate/amplify tics in susceptible individuals. | Dietl T et al.(10) |
|  |  | Case report | Negative (exacerbated) | An autistic child developed recurrence/worsening of motor tics during acute oral prednisolone therapy, despite concurrent aripiprazole and clonidine. | Figueiredo T et al.(11) |
|  | Corticosteroid therapy (agent not specified) | Case report (early brief report) | Positive (improved) | TS improvement after corticosteroid therapy was reported (very low-level evidence; small sample; best interpreted as hypothesis-generating). | Kondo K, Kabasawa T et al.(12) |
|  | ACTH + prednisone | Case report (2 cases) | Positive (improved/remitted) | Two TS cases with infection-related features and poor response/intolerance to neuroleptics improved markedly after ACTH + prednisone; suggests benefit in a potential immune/inflammation-driven subgroup. | Matarazzo EB et al.(13) |
| **Anti-IgE monoclonal antibody** | Omalizumab | Case series (subgroup within n=27) | Positive (improved/remitted; often combined therapy) | Overall, standardized AR management over 2–6 months reduced YGTSS from 6.8 ± 1.4 to 3.5 ± 0.7; 12/27 received omalizumab. A representative case (300 mg monthly) showed resolution of tics and hyperactivity after 4 injections and sustained control after 14 injections. | Yu RL et al.(4) |

**Reference：**

1. Gober HJ, Li KH, Yan K, Bailey AJ, Carleton BC. Hydroxyzine Use in Preschool Children and Its Effect on Neurodevelopment: A Population-Based Longitudinal Study. Front Psychiatry. 2021;12:721875.

2. Akinsiku F. Tics induced by promethazine in an adolescent: a case report. BJPsych Open. 2021;7(S1):S113-S4.

3. Simons FE. Prospective, long-term safety evaluation of the H1-receptor antagonist cetirizine in very young children with atopic dermatitis. ETAC Study Group. Early Treatment of the Atopic Child. J Allergy Clin Immunol. 1999;104(2 Pt 1):433-40.

4. Yu RL, Wang J, Wang XS, Wang HT, Wang XY. Management of allergic rhinitis improves clinical outcomes of difficult-to-treat tic disorders or attention-deficit/hyperactivity disorders. Allergol Select. 2023;7:191-7.

5. Tsai ML, Lin HC, Yen CH, Ku JT, Sung SY, Chang H. Increased Risk of Tourette Syndrome with Leukotriene Modifier Use in Children with Allergic Diseases and Asthma: A Nationwide Population-Based Study. Children (Basel). 2022;9(11).

6. Lei WT, Lin CY, Chu SH, Fang LC, Kao YH, Tsai PL, et al. The Impact of Montelukast Duration on the Risk of Neuropsychiatric Disorders in Children with Asthma: A Population-Based Cohort Study. Pharmaceuticals (Basel). 2025;18(3).

7. Trau SP. An Unusual Case of New Tic Emergence and Exacerbation Following Treatment With Fluticasone Propionate. Pediatr Neurol. 2020;105:67-8.

8. Steele M, Rosner J. A possible link between fluticasone propionate and tics in pediatric asthmatics. Can J Neurol Sci. 2012;39(6):851.

9. D'Silva N, Lindsay D. M052 MOTOR TICS, AN UNKNOWN SIDE EFFECT OF INTRANASAL FLUTICASONE. Annals of Allergy, Asthma & Immunology. 2021;127(5):S73.

10. Dietl T, Kumpfel T, Hinze-Selch D, Trenkwalder C, Lechner C. [Exacerbation of tics by prednisolone]. Nervenarzt. 1998;69(12):1111-4.

11. Figueiredo T. The Recurrence of Motor Tics Mediated by Oral Prednisolone Use in Autistic Children: A Case Report. Clin Neuropharmacol. 2021;44(4):145-7.

12. Kondo K, Kabasawa T. Improvement in Gilles de la Tourette syndrome after corticosteroid therapy. Ann Neurol. 1978;4(4):387.

13. Matarazzo EB. Tourette's Syndrome Treated with ACTH and Prednisone: Report of Two Cases. J Child Adolesc Psychopharmacol. 1992;2(3):215-26.
